# Cortical thickness and subcortical structure volume abnormalities in patients with major depression with and without anxious symptoms

**DOI:** 10.1002/brb3.754

**Published:** 2017-06-27

**Authors:** Ke Zhao, Haiyan Liu, Rui Yan, Lingling Hua, Yu Chen, Jiabo Shi, Qing Lu, Zhijian Yao

**Affiliations:** ^1^ Department of Psychiatry Affiliated Nanjing Brain Hospital of Nanjing Medical University Nanjing China; ^2^ Research Center of Learning Science Southeast University Nanjing China; ^3^ Medical School Nanjing University Nanjing China

**Keywords:** anxiety, biological markers, brain imaging, depression, mood disorders

## Abstract

**Background:**

Anxious depression is one of the common subtypes of major depressive disorder (MDD). Clinically, patients with anxious depression exhibit more severe depressive symptoms than patients with nonanxious depression. The aim of the present study was to explore the common and differing cortical and subcortical structural changes between patients with anxious and nonanxious depression.

**Methods:**

Patients were placed into one of three groups: the anxious depression group (MDD patients with high levels of anxiety symptoms, *n* = 23), the nonanxious depression group (*n* = 22), and healthy controls (*n* = 43) that were matched for age, sex, and education level. All participants underwent T1‐weighted MRI. The Freesurfer, which uses a set of automated sequences to analyze the abnormal changes of cortical thickness, cortical and subcortical structures, was used to process the T1 images.

**Results:**

Compared to controls, MDD patients showed thinner cortical thickness in the left inferior temporal, the right superior temporal, and the right parsorbitalis, and a smaller volume of the left hippocampus. Compared to nonanxious depression, anxious depressive patients showed a cortical thinning of the left superior frontal and right superior temporal, as well as the right lingual, and significantly increased subcortical volume of the bilateral caudate nuclei. Correlation analysis showed that the volumes of the bilateral caudate nuclei were directly proportional to the anxiety/somatization factor score.

**Conclusions:**

These findings suggest that smaller hippocampal volume and atrophic prefrontal and temporal cortices might be a common pattern of cortical and subcortical alterations in patients with depression and/or anxiety. However, the change in the caudate nucleus volume may be indicative of anxious depression and may potentially be used to distinguish anxious from nonanxious depression.

## 1 INTRODUCTION

Anxious depression is one of the common and clinically relevant subtypes of major depressive disorder (MDD; Rush, 2007). The DSM‐V requires clinicians to complete an “anxiety specifier” that helps to distinguish depressed individuals with accompanying anxious symptoms from those without anxiety (Goldberg et al., 2014). Indeed, it has been estimated that 40–50% of MDD patients, whether inpatient or outpatient, have at least one comorbid anxiety disorder (Fava et al., 2006; Wiethoff et al., 2010). Clinically, compared to nonanxious depressive patients, anxious depression presents with more frequent depressive episodes, more severe symptoms and side‐effects, and patients exhibit worse outcomes and treatment responses (Fava et al., 2004, 2008). Unipolar and bipolar depression accompanied by anxious symptoms has a worse outcome and a higher frequency of depressive episodes than depression without anxiety (Goes, 2015; Goldberg & Fawcett, 2012). Furthermore, anxious depressive patients take twice as long to recover from depressive episodes and are more prone to physical discomfort, depersonalization, derealization, and a higher proportion of suicide attempts than patients without anxiety symptoms (Fava et al., 2004; Goldberg, 2014).

Previous studies found that high trait anxiety was a vulnerability factor for MDD (Sandi & Richter‐Levin, 2009), and that there might be a common mechanism for the development of anxiety and depression. The two disorders have been related to a dysfunction of the hypothalamic–pituitary–adrenocortical (HPA) axis, and recent studies suggest that the stress response and hormone imbalances, such as cortisol hypersecretion, can cause a series of neurological dysfunctions that may be related to the pathogenesis of anxiety and depression (Camacho, 2013; Leonard & Myint, 2009). However, two diseases have different characteristics. From a psychology perspective, depression is characterized by a lack of positive affectivity, and physiological hyperarousal is a feature of anxiety, whereas comorbid anxiety and depression show a high level of negative affectivity (Clark & Watson, 1991). Two neuroimaging studies based on symptoms found that depression and anxiety symptoms in relation to connectivity patterns and especially show the differential patterns in emotion networks and cognitive control networks (Oathes, Patenaude, Schatzberg, & Etkin, 2015; Spielberg et al., 2014). Previous study found that on Parametric Go/No‐Go task, anxious depression had more activation in the anterior dorsolateral prefrontal cortex, hippocampus, and caudate during rejections, and inferior parietal lobule during correct Targets than MDD (Crane et al., 2016). This result suggested that hypervigilance in individuals with MDD comorbid with anxiety within the cognitive control network.

Functional imaging results show anxious depression and nonanxious depression have different characteristics in the emotion and cognitive control systems. However, little is known about its brain structure basis. Few studies have attempted to explore brain structure changes in patients with anxious depression, which may have a unique pathological mechanism. One structural MRI study compared four groups of patients with MDD, panic disorder, social anxiety disorder, and comorbid MDD and anxiety. There were reduced volumes of the rostral anterior cingulate cortex in the four patient groups compared with the healthy controls, but compared with the other subgroups, comorbid anxiety and depression did not lead to significant changes in gray matter volume (van Tol et al., 2010). Another study reported that 96 patients with MDD had lower gray matter volume in the right superior and inferior temporal gyrus compared to 49 anxious depression patients (Inkster et al., 2011). The latest structural MRI study showed that MDD patients with generalized anxiety disorder comorbidity showed thinner cortical thicknesses in the medial orbitofrontal and fusiform gyri in the right hemisphere, and in the temporal pole and lateral occipital cortices in the left hemisphere compared with healthy controls and patients with nonanxious depression (Canu et al., 2015). These results suggest a possible diagnosis‐dependent brain structure alteration in anxious depression. Notably, these studies defined anxious depression with inconsistent standards, which makes comparing these results difficult. For this reason, we used a dimensional definition of DSM‐diagnosed MDD with a score of no <7 for anxiety/somatization factor of HAM‐D 17 to separate anxious depression from nonanxious depression. This definition has been recommended for the differentiation of anxious depression as a clinical subtype and is useful to explore the changes in the brain structure of anxious depression (Ionescu et al., 2013). In our study, we compared cortical thickness and cortical and subcortical volumes between anxious and nonanxious depression. Combined with previous functional and structural findings, we hypothesized that compared with healthy controls, anxious depression and nonanxious depression would exhibit similarities gray matter change at limbic system which is an important circuit of emotion regulation, and differences gray matter change in the circuit of cognitive control.

## 2 METHODS

### 2.1 Participants and assessments

This study included 45 inpatients with MDD from the Affiliated Nanjing Brain Hospital of Nanjing Medical University and 43 healthy subjects who were matched for age, sex, and education years. The MDD patients were aged from 20 to 45 years. Depression diagnoses were confirmed by a psychiatrist using the Structured Clinical Interview for DSM‐IV Axis I disorders (SCID), and the healthy controls were also used the Structured Clinical Interview for DSM‐IV Axis I disorders —Research version—Non‐Patient Edition (SCID‐I/NP) to confirm their status. All patients had a score of at least 14 on the Hamilton Rating Scale for Depression (HAM‐D) 17‐item scale on the day of scanning. The patient group was divided into two subgroups: the anxious depression subgroup included 23 MDD patients with a score of ≥7 on the anxiety/somatization factor score of HAM‐D 17 (Ionescu, Niciu, Henter, & Zarate, 2013; Ionescu et al., 2013), and the nonanxious subgroup who had a score of <7 on the HAM‐D 17 scale. The anxiety/somatization factor of the HAM‐D 17 scale includes six items: psychic anxiety, somatization anxiety, gastrointestinal somatic symptoms, general somatic symptoms, hypochondriasis and insight (Sharp, 2015). The exclusion criteria for the patients were as follows: (1) combined with other psychiatric or neurological illness (e.g., head trauma, mental development deficit, psychotic symptoms orepilepsy); (2) history of substance abuse and alcohol dependence; (3) electric shock therapy within 2 weeks; (4) pregnant and lactating women; (5) physical contraindications for MRI. Healthy controls and their first‐degree relatives had no history of psychiatric illness.

The usage of antipsychotic drugs: 25 patients were first episode, and had never been medicated (11 patients were anxious depression and 14 patients were nonanxious depression); eight patients had been treated with medication, and did not take drugs in the last 3 months (five patients were anxious depression and three patients were nonanxious depression); 12 patients were being treated with drugs (seven anxious depression patients were all taking SSRI, three nonanxious depression patients were taking SSRI drugs, and two nonanxious depression patients were taking SNRI).

All participants were informed of the study and provided written informed consent. This study was approved by the Research Ethics Review Board.

### 2.2 MRI data acquisition

Three‐dimensional anatomical T1‐weighted MRI images were acquired from a 3.0‐T Siemens verio scanner with an eight‐channel radio frequency coil. Subjects were positioned comfortably in the coil and fitted with soft earplugs. Throughout the scan subjects were instructed to relax and remain still. High‐resolution 3D T1‐weighted data were acquired with the following parameters: TR = 1900 ms, TE = 2.48 ms, flip angle = 9°, 176 axial slices with thickness = 1 mm, in plane voxel resolution = 1 × 1 mm, FOV = 25 × 25 cm^2^, acquisition time = 4 min 18 s. All of the images were checked by a doctor of imaging to rule out gross structural abnormalities.

### 2.3 Cortical thickness analysis

The T1 images were processed using the Freesurfer package 5.3.0 (http://surfer.nmr.mgh.harvard.edu), which uses a set of automated sequences to reconstruct the cortical surface, and cortical thickness of the whole brain was measured (Dale, Fischl, & Sereno, 1999; Fischl & Dale, 2000). The whole process was completed automatically, including motion correction, skull stripping, segmentation of white matter, creation of the pial surface and surface of the white/gray junction, inflation of the folding surface plane and topology correction (Fischl, Sereno, & Dale, 1999; Fischl et al., 2002; Segonne et al., 2004). Next, a manual method was used to correct any geometric inaccuracies. The shortest distance between the pial surface and the white/gray junction is the cortical thickness at each point. In order to make a comparison between groups, all of the corrected cortical thickness maps were generated a common average surface and were smoothed using a Gaussian kernel of 15 mm full‐width half‐maximum, which was prepared for group statistical analysis.

### 2.4 Cortical and subcortical volume analysis

The cortical and subcortical volumes were also measured automatically using the FreeSurfer software. The final segmentation was based on a subject‐independent probabilistic atlas and subject‐specific measured values. The atlas, which was a manually labeled training set, was mapped into Talairach space in which all the subjects’ images were registered, and then the value for each voxel was measured and labeled (Fischl et al., 2004). A more detailed explanation of the technical aspects can be found in (Dale et al., 1999; Fischl et al., 2002, 2004). There were 33 regions in the entire cortex of each hemisphere and six subcortical structures (hippocampus, amygdala, caudate, thalamus, pallidum, and putamen) that were closely related to emotional regulation. The total intracranial volume (ICV), which was also calculated using Freesurfer, was described in the previous study (Buckner et al., 2004).

### 2.5 Statistical analyses

Group differences in the basic demographics were examined with two‐tailed Student *t* tests for continuous variables (age, education, duration of illness, and HAM‐D 17 scores) and chi‐square tests were used for categorical variables (sex)using the Statistical Product and Service Solutions (SPSS) 19.0 software.

Freesurfer's Qdec (http://surfer.nmr.mgh.harvard.edu/fswiki/FsTutorial/QdecGroupAnalysis_freeview) used a general linear model (GLM) to estimate the differences in the cortical thickness at each vertex of the surface between the MDD group and the control group, and between the two subgroups of MDD with age, years of education and sex as covariates. Because all the subjects’ cortical thickness maps were aligned to a common surface template, the ICV was not used as a covariate. Monte Carlo permutation cluster analysis (*p *<* *.05) with 10,000 permutations was applied for multiple comparisons correction.

Cortical and subcortical volumes were automatically derived outcomes from Freesurfer. The GLM in SPSS 19.0 was used to analyze the differences in volumes between groups, and the age, sex, education years, and ICV were controlled as covariates. A significance level of *p *<* *.008 (Bonferroni multiple comparisons correction *p *<* *.05/6) was assumed for the analysis of six subcortical volumes in each hemisphere, and a significance level of *p *<* *.0015 (Bonferroni multiple comparisons correction *p *<* *.05/33) for the analysis volume of 33 cortical regions in each hemisphere.

## 3 RESULTS

### 3.1 Sample characteristics

Demographic and clinical features are shown in Table 1, including the terms of age, sex, education years, and HAM‐D 17 scores of healthy controls and MDD patients, and terms of number of episodes and duration of illness of MDD patients. The patient group and the healthy control group did not differ significantly in terms of age, sex or years of education, and the two subgroups (anxious vs. nonanxious) did not differ significantly in terms of duration of illness or number of episodes. However, HAM‐D 17 scores were significantly decreased in the nonanxious depression group compared to the anxious group, due to the significantly lower scores of the anxiety/somatization factor on the HAM‐D 17 scale.

**Table 1:** Sample demographics

	Healthy control subjects (*n* = 43)	MDD subjects (*n* = 45)	*p* value	Anxious depression (*n* = 23)	Nonanxious depression (*n* = 22)	*p* value
Female (%)	21 (49%)	20 (44%)	.41	10 (43%)	10 (45%)	.56
Age (years)	31.28 ± 7.81	32.69 ± 7.85	.40	32.13 ± 8.24	33.27 ± 7.55	.63
% Han	100%	100%	–	–	–	–
Years of education	14.9 ± 2.10	13.87 ± 3.20	.07	14.65 ± 3.56	13.05 ± 2.59	.09
% Right handedness	100%	100%	–	–	–	–
HAM‐D (17‐item)	0.63 ± 1.05	24.89 ± 5.51	<.001	27.57 ± 4.99	22.09 ± 4.65	<.001
HAM‐D without anxiety/somatization factor	0.42 ± 0.66	18.49 ± 4.38	<.001	19.30 ± 4.54	17.63 ± 4.14	.205
Illness duration (months)	–	6.27 ± 4.06	–	6.57 ± 4.14	5.95 ± 4.04	.62
Number of episodes	–	2.00 ± 1.50	–	1.96 ± 1.40	2.05 ± 1.65	.85

MDD, major depressive disorder; HAM‐D, Hamilton Depression Rating Scale; HAM‐D‐anxiety/somatization factor, HAM‐D total score minus anxiety/somatization factor.

### 3.2 Cortical thickness analysis

**Figure 1:**
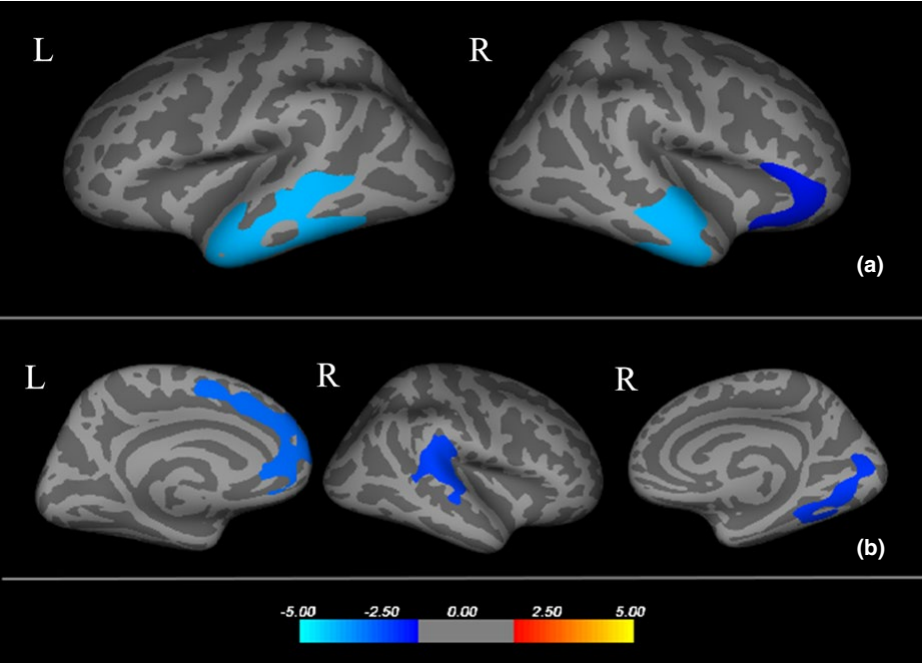
(a) Three areas with thinner cortical thickness in major depressive disorder (MDD) patients compared with healthy controls, respectively, Left inferior temporal, Right superior temporal and Right pars orbitalis. (b) Three areas with thinner cortical thickness in anxious depression patients compared with nonanxious depression patients, respectively, Left superior frontal, Right lingual and Right superior temporal. Cooler colors(negative values) represent significant cortical thinning and warmer colors (positive values) represent significant cortical thickening. The color‐coding for *p‐*values is on a logarithmic scale of ‐1‐ ‐5. L, left hemisphere; R, right hemisphere

Compared to controls, three clusters were significantly thinner in the MDD patients (*p *<* *.05 after Monte Carlo permutation correction), including thinner cortical thickness in the left inferior temporal, and right superior temporal, as well as in the right pars orbitalis. No regions with significantly greater cortical thickness were found in MDD patients. When comparing subgroups, anxious depressive patients showed a thinner cortical thickness in the left superior frontal, right superior temporal, and right lingual lobes. No region with significantly greater cortical thickness were found in anxious depression patients. (Figure 1 and Table 2).

**Table 2:** Areas with cortical thickness differences between healthy controls and MDD patients, as well as between two subgroups in MDD

				Talairach coordinates^a^			
MDD versus HC^b^	MDD (mm)^c^	HC (mm)^c^	Size (mm^2^)	*x*	*y*	*z*	CWP
Left inferior temporal	2.76 ± 0.12	2.99 ± 0.11	5085.76	−49.0	−42.6	−14.7	0.0002
Right superior temporal	2.48 ± 0.13	2.87 ± 0.15	3428.99	59.9	−3.2	−4.5	0.0001
Right pars orbitalis	2.56 ± 0.19	2.68 ± 0.15	1661.52	44.2	35.9	−8.8	0.0192
Anxious depression versus Nonanxious depression^b^	Anxious depression (mm)	Nonanxious depression (mm)					
Left superior frontal	2.85 ± 0.16	3.01 ± 0.14	2259.39	−9.1	31.4	27.3	0.0012
Right superior temporal	2.38 ± 0.15	2.54 ± 0.09	2006.54	55.7	−28.7	9.0	0.0048
Right lingual	2.18 ± 0.20	2.29 ± 0.22	1923.06	22.5	−69.9	−1.1	0.0064

MDD, major depressive disorder; CWP, cluster‐wise *p*‐value.

a Coordinates of the maximum voxel for the cluster.

b Age and sex as covariates.

c Regional mean cortical thickness.

### 3.3 Cortical and subcortical volume analysis

**Figure 2:**
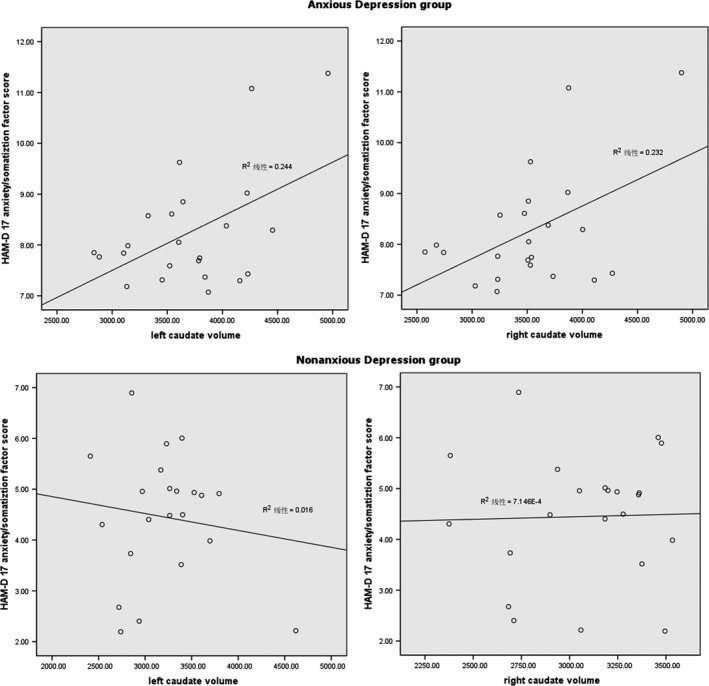
The correlation between the volume of bilateral caudates and the Hamilton rating scale for depression (HAM‐D) 17 anxiety/somatization factor score; age, gender, education years and intracranial volume (ICV) were controlled as covariates

There was a cortical and subcortical volume reduction in the left hippocampus in the MDD group, and the patients with anxious depression exhibited significantly increased subcortical volume in the bilateral caudates compared to those with nonanxious depression. All the corrected and uncorrected results are shown in Table 3. Pearson partial correlation analysis was performed between the bilateral caudates in both subgroups and the HAM‐D 17 anxiety/somatization factor score; age, gender, education years, and ICV were controlled as covariates. In the anxious depression group, the volumes of the left caudate(*r* = .494, *p *=* *.027) and right caudate(*r* = .482, *p *=* *.031) were directly proportional to the anxiety/somatization factor score. However, there was no correlation between anxiety/somatization factor score and the caudate volumes in the nonanxious depression group (Figure 2).

**Table 3:** Cortical and subcortical structure volume differences between anxious depression group and nonanxious group, as well as between MDD group and healthy control group

Cortical and subcortical regions	Volume (mm^3^), Mean ± *SD*		General linear model (GLM)	
	MDD subjects	Healthy control subjects	*F*	*p* value
Left hippocampus	4472.42 ± 443.65	4769.72 ± 388.90	9.36	.003^a^
Right hippocampus	4659.64 ± 431.62	4872.80 ± 351.74	5.70	.019
Left pars opercularis gyrus	4527.26 ± 621.54	4950.14 ± 859.41	9.34	.003
Left superior frontal gyrus	21223.19 ± 2595.84	22595.14 ± 2328.89	8.65	.004
Left gyrus	3199.52 ± 443.73	3367.33 ± 488.75	6.43	.013
Right superior temporal gyrus	11375.58 ± 1335.13	12067.10 ± 1452.31	5.77	.019
Right frontal pole gyrus	1011.30 ± 160.33	1088.88 ± 161.59	5.31	.024

	Anxious depression subjects	Nonanxious depression subjects	*F*	*p* value
Left caudate	3713.57 ± 541.21	3215.80 ± 513.98	8.33	.006^a^
Left putamen	6012.87 ± 532.67	5546.15 ± 711.58	4.79	.035
Left pallidum	1509.96 ± 162.25	1340.78 ± 222.32	6.89	.012
Right caudate	3522.17 ± 540.89	3076.12 ± 375.31	9.26	.004^a^
Left transverse temporal gyrus	1182.59 ± 181.28	1053.10 ± 156.17	4.16	.049
Right lingual gyrus	6367.00 ± 740.76	6729.24 ± 849.46	4.39	.043

MDD, major depressive disorder; *SD*, standard deviation.

a Regions remains significant after controlling for multiple comparisons at the level of the six different subcortical structures in each hemisphere using Bonferroni correction (*p* < .05/6 = .008).

## 4 DISCUSSION

A comprehensive analysis of gray matter structure can determine changes in the whole brain structure in anxious depression patients and may find structural neuroimaging biomarkers to help differentiate anxious depression from other subtypes. In our study, we explored the common and differing cortical thickness and gray matter volume changes between anxious and nonanxious depression patients.

Our first finding was that the MDD group had significantly reduced cortical and subcortical volumes of the left hippocampus and thinner cortical thickness in the left inferior temporal and right superior temporal, as well as in the right pars orbitalis, compared with healthy controls. These abnormalities appear to be common trait changes of anxious and nonanxious depressive patients. The hippocampus is one of the most studied limbic structures and is considered to be the integrator of emotion and cognition, and it is closely related to the formation of new memories and guides behavior by comparing new stimulatory input to stored memories (Bracht et al., 2014; Crane et al., 2016). Previous studies have reported that the volume of the hippocampus is reduced in both MDD and anxiety disorders (Campbell, Marriott, Nahmias, & MacQueen, 2004; Jacobs et al., 2014; Lamers et al., 2016; Oathes et al., 2015), and a reduction of the hippocampal volume has been related to the severity and duration of the depression disorder (Bell‐McGinty et al., 2002; MacQueen et al., 2003). The hippocampus is the upper regulation central of the hypothalamic–pituitary–adrenal (HPA) axis, which provides negative feedback to the hypothalamic corticotropin‐releasing factor release through glucocorticoid action (Winograd‐Gurvich et al., 2006). Individuals with mood disorders show HPA over activity and increased glucocorticoid levels, and some studies have suggested that the smaller hippocampus in patients with mood disorders is related to dysfunction of the HPA axis (McEwen, 2007; Vreeburg et al., 2009). Based on the above, we postulate that smaller hippocampal volume might be a common brain structural change in patients with depression and/or anxiety.

Compared to healthy controls, depressed patients exhibited thinner cortical thickness in the left inferior temporal, and right superior temporal, as well as in the right pars orbitalis. Our results are supported by previous studies that found a prefrontal and temporal cortex alteration in both anxiety and depressed patients (Canu et al., 2015; Ducharme et al., 2014; Murray, Wise, & Drevets, 2011). The prefrontal cortex is divided into ventromedial and dorsolateral areas, and the two parts are closely related to mood disorders. The ventromedial cortex of the prefrontal lobe is involved in emotion processing and motivation, and the dorsolateral prefrontal cortex is associated with cognitive processing (Brzezicka, 2013; Shang et al., 2014). The inferior temporal and superior temporal cortex are associated with the prefrontal cortex and are related to sensory integration, introspective functions, and visceral reactions to emotional stimuli (Drevets, Price, & Furey, 2008). Notably, compared to nonanxious depression, anxious depressive patients showed thinner cortical thickness in the prefrontal, temporal and lingual lobes, which is consistent with previous studies (Canu et al., 2015). We speculate that atrophic prefrontal and temporal cortices affect the normal emotional processing in patients with high levels of anxiety and those showing fear disinhibition and over reaction to stimuli. Combined with clinical symptoms, anxious depressive patients exhibit more severe depressive symptoms, higher suicide rates, and worse outcomes and treatment response than those with nonanxious depression, which might be related to more severe brain structural damage in the emotional–cognitive neural circuits.

Our second finding was that the patients with anxious depression exhibited significantly increased subcortical volumes in the bilateral caudate and thinner cortical thickness in the left superior frontal, right superior temporal, and right lingual lobes compared with those with nonanxious depression. Frontal lobe and temporal lobe are involved in cognitive regulation (Quinn et al., 2012). The caudate nucleus also closely link with cognitive control, which working together with the dorsal prefrontal cortex involved in working memory and strategic planning processes (Haber, 2016). Previous fMRI study found that compared MDD and healthy controls, comorbidity of depression and anxiety had more activation in the caudate during rejections part of Parametric Go/No‐Go task (Crane et al., 2016). A structure study reported that subjects with anxiety disorder showed larger right striatum volume compared with controls, and the worry severity and volume of the left caudate nucleus positively correlated (Hilbert et al., 2015). These results suggested that caudate nucleus was more active in patients with anxiety, and closely related with physiological hyperarousal of anxiety. Some researchers believed that the differential patterns of the network may be present based upon the with or without anxiety symptoms in depression (Crane et al., 2016; Jacobs et al., 2014). The deeper study found that volume of caudate nucleus is positively associated with HAM‐D 17 anxiety/somatization factor score in patient with anxious depression, but not in patient with nonanxious depression. These results suggested that caudate nucleus volume was directly related to anxiety and somatization symptoms in anxious depression patient, and the volume of caudate nucleus might be an important characteristic to identify anxious depression to other subtypes in depression.

There are some limitations to our study. Firstly, the sample size was relatively small. Although we have carried out rigorous multiple corrections of the results, it will be necessary to study a larger sample size in future to reduce the type II error rate. Secondly, in our study, the regions with thinner cortical thickness did not show a smaller cortical volume. In contrast to our findings, a previous study reported a reduced volume of the right temporal lobe in nonanxious depression compared with anxious depression (Inkster et al., 2011). However, this study is different from the brain imaging analytical methods we used, and the definition of anxious depression was also different from our study. On the other hand, some researchers have proposed that cortical volume is mainly affected by cortical surface area rather than cortical thickness (Im et al., 2008; Winkler et al., 2010). Nevertheless, some results of cortical volume changes in our study failed to stay significant with correction, and this be should be tested in future studies. In our studies, we did not find significant changes in the volume of the amygdala in the patient group compared with the healthy controls. The volumes of the amygdala may vary in relation to the illness duration and state; some studies found that MDD patients who were treatment‐naïve and those with a longer illness duration tended to show amygdala volumetric reductions while increased amygdala volumes were found in patients earlier in the course of the illness (Hamilton, Siemer, & Gotlib, 2008). In our study, the illness duration was relatively short, and some participants were treated with antidepressants, which might have an effect on the gray matter volume changes. Future research may choose the first‐episode patients to avoid the effect of drug treatment.

## 5 CONCLUSION

We observed volume alterations in the left hippocampus and thinner cortical thickness in the prefrontal and temporal lobes in MDD patients compared to healthy controls, and this result suggests that anxious depression and nonanxious depression patients share a common pattern of cortical and subcortical alterations. However, anxious depressive patients showed thinner cortical thickness in the prefrontal and temporal cortices and increased volume of caudate, which may prompt more serious dysfunction in cognitive regulation circuits than that in nonanxious depression. Additionally, the volume of the bilateral caudate nucleus was positively associated with HAM‐D 17 anxiety/somatization factor score in patients with anxious depression. Our findings suggest that the change in the caudate nucleus volume may be a possible neuroimaging marker of anxious depression and could be used to distinguish anxious from nonanxious depression.

## CONFLICT OF INTEREST

The authors declare no conflict of interest.
